# Bacterial communities in the rumen of Holstein heifers differ when fed orchardgrass as pasture vs. hay

**DOI:** 10.3389/fmicb.2014.00689

**Published:** 2014-12-09

**Authors:** Riazuddin Mohammed, Geoffrey E. Brink, David M. Stevenson, Anthony P. Neumann, Karen A. Beauchemin, Garret Suen, Paul J. Weimer

**Affiliations:** ^1^Lethbridge Research Center, Agriculture and Agri-Food CanadaLethbridge, AB, Canada; ^2^US Department of Agriculture, United States Dairy Forage Research Center, Agricultural Research ServiceMadison, WI, USA; ^3^Department of Bacteriology, University of WisconsinMadison, WI, USA

**Keywords:** rumen microbiology, bacterial community composition, pasture, hay, forage, volatile fatty acids

## Abstract

The rich and diverse microbiota of the rumen provides ruminant animals the capacity to utilize highly fibrous feedstuffs as their energy source, but there is surprisingly little information on the composition of the microbiome of ruminants fed all-forage diets, despite the importance of such agricultural production systems worldwide. In three 28-day periods, three ruminally-cannulated Holstein heifers sequentially grazed orchardgrass pasture (OP), then were fed orchardgrass hay (OH), then returned to OP. These heifers displayed greater shifts in ruminal bacterial community composition (determined by automated ribosomal intergenic spacer analysis and by pyrotag sequencing of 16S rRNA genes) than did two other heifers maintained 84 d on the same OP. Phyla Firmicutes and Bacteroidetes dominated all ruminal samples, and quantitative PCR indicated that members of the genus *Prevotella* averaged 23% of the 16S rRNA gene copies, well below levels previously reported with cows fed total mixed rations. Differences in bacterial community composition and ruminal volatile fatty acid (VFA) profiles were observed between the OP and OH despite similarities in gross chemical composition. Compared to OP, feeding OH increased the molar proportion of ruminal acetate (*P* = 0.02) and decreased the proportion of ruminal butyrate (*P* < 0.01), branched-chain VFA (*P* < 0.01) and the relative population size of the abundant genus *Butyrivibrio* (*P* < 0.001), as determined by pyrotag sequencing. Despite the low numbers of animals examined, the observed changes in VFA profile in the rumens of heifers on OP vs. OH are consistent with the shifts in *Butyrivibrio* abundance and its known physiology as a butyrate producer that ferments both carbohydrates and proteins.

## Introduction

Ruminal microbes, particularly bacteria, perform a multitude of functions in the process of digesting dietary organic matter (Russell, 2002). They play an essential role in fermenting simple and complex carbohydrates into volatile fatty acids (VFA); in the biohydrogenation of polyunsaturated fatty acids; and in the degradation of dietary protein and partial recapture of non-protein nitrogen as microbial cell protein for subsequent protein nutrition of the animal. It is well known that ruminal bacterial communities respond to changes in diet and environmental conditions (Dehority and Orpin, 1997; Dehority, 2003) and also exhibit host specificity (Weimer et al., 2010a). Most of the studies that have examined the influence of diet on ruminal bacterial communities have employed total mixed rations (TMR) containing various proportions of forage and concentrates such as cereal grains (Boeckaert et al., 2008; Kim et al., 2008; Pitta et al., 2014). Few studies have compared ruminal bacterial communities on all-forage diets, despite the fact that much of the world's ruminant production is based on such diets. In particular, there are no reports to our knowledge that have compared ruminal bacterial communities between animals fed fresh vs. conserved forages (hay or silage) in forage-only diets.

This study was initiated to examine the effect of feeding Holstein heifers orchardgrass pasture (OP) or orchardgrass hay (OH) on ruminal bacterial community composition (BCC) characterized by automated ribosomal intergenic spacer analysis (ARISA) to compare whole communities, and by pyrotag sequencing of 16S rRNA genes to make more specific comparisons at the phylum and genus levels. Heifers were used rather than lactating cows because the distance of our grazing plots from our milking parlor (~3 km, across a state highway) precluded incorporation of a lactation study. Because the study was conducted over the summer months when quality of orchardgrass and other cool-season grasses in the upper Midwestern USA varies substantially (Brink and Casler, 2012; Brink et al., 2013), an experimental design was selected in which one group of 3 heifers was alternately grazed OP (period 1), then fed OH harvested from the same field at the same growth stage (period 2), then returned to OP (period 3). For comparison, a group of 2 heifers was maintained on OP for all 3 periods. Each period was 28 d in length. Based on expected changes in chemical composition for forage during harvest and preservation as hay, we hypothesized that BCC would differ among heifers grazing OP compared with those fed OH, and that these differences would be associated with differences in forage composition and/or differences in the profiles of primary fermentation products (VFA ratios) in ruminal fluid.

## Materials and methods

### Pasture and hay feeding of heifers

Experiments were conducted during June-August 2009 at the US Dairy Forage Research Center Farm near Prairie du Sac, WI, USA (43°20′24″N-89°43′12″W), in strict accordance with the recommendations in the Guide for the Care and Use of Laboratory Animals of the National Institutes of Health, under research protocol A01427 approved by the University of Wisconsin-Madison College of Agricultural and Life Sciences Animal Care and Use Committee. Ruminal cannulations were performed under a separate protocol (A01307) by a licensed University of Wisconsin veterinarian, using lidocaine anesthetic to minimize animal discomfort. Five ruminally cannulated Holstein heifers (mean age 17 ± 0.89 mo; mean BW 465 ± 14 kg) were fed orchardgrass (*Dactylis glomerata* cv “Bronc”) as either pasture (OP) or hay (OH). Of the 5 heifers, 3 were offered OP, OH, and OP during the 1st, 2nd, and 3rd periods, respectively, while the other 2 heifers remained on OP in all three periods. This experimental design was chosen because quality and composition of cool-season grasses such as orchardgrass varies dramatically during the summer in the upper Midwestern USA (Brink et al., 2013), making a switchback design inappropriate. Alternative designs involving a comparison of groups of animals fed only pasture vs. those fed only hay were rejected because they would not allow detection of diet-dependent shifts in BCC or in ruminal chemistry within individual animals.

The duration of each experimental period was 28 days. During each period, two pastures of 0.6 and 0.4 ha were each subdivided into 0.1 ha paddocks. Beginning with the paddock having the grass at v2–v4 stage (residual sward height of 10 cm; Moore et al., 1991) heifers grazed for 2 days before being moved to the next paddock. Heifers returned to the first paddock after completing one rotation of all the paddocks. Pasture herbage allowance was at least three times the anticipated dry matter (DM) intake per animal (11 kg DM d^−1^). For making OH, orchardgrass was harvested at the same maturity stage from a paddock adjacent to the grazing pastures. The grass was wilted for 48 h after cutting, raked, and further dried to 20% moisture over 24 h before being baled in small square bales (approximately 20 kg each). OH was fed *ad libitum* from racks in an unvegetated corral adjacent to the pasture plots. All heifers had access to water and were kept outdoors for the entire period of the study.

Specific measurements of intake or animal movement were not made, but the combination of probably low intakes by young, non-lactating animals and lush pasture growth allowed us to use small paddocks. The resulting limited movement of the animals probably did not result in significant differences in energy expenditure among the two dietary groups.

### Forage analysis

The pastures grazed by the heifers were sampled by clipping the grass from three randomly-placed quadrats (metal squares 25 × 100 cm) to a 10-cm residual sward height and then dried at 55°C. The forage sample representing each period was a composite of grass samples collected from each paddock grazed within a rotation. Although the OP samples were dried relatively rapidly in a forced-air oven, minor changes in nutrient composition due to plant cellular respiration may have occurred within the first few hours. Duplicate samples of dried OP and OH were ground through a 1-mm screen of a Wiley mill, and were analyzed for crude protein (CP; Leco FP-2000 A nitrogen analyzer, Leco Corp., St. Joseph, MI), neutral detergent fiber (NDF; using the procedure of Mertens, 2002 to include both α-amylase and sodium sulfite during refluxing), as well as acid detergent fiber (ADF) and acid detergent lignin (Van Soest et al., 1991). Water-soluble carbohydrates (WSC) were determined by colorimetric analysis of aqueous extracts. In brief, 1 g of oven-dried (55°C) forage sample was suspended in 40 mL of deionized water (pre-warmed to 39°C) and shaken in a reciprocal shaker (120 cycles min^−1^) for 60 min in a room maintained at 39°C. The aqueous extracts were then centrifuged at 10,000 × g for 5 min at room temperature, and a subsample of the supernatant (25 μL) was analyzed in duplicate by the phenol-sulfuric acid method (Dubois et al., 1956), using glucose as standard.

### Ruminal samples and processing

Ruminal digesta were collected at ~13:00 h on 3 consecutive days of each period (days 26–28) by spot sampling from the midpoint along the length of the ruminal contents and from the midpoint along the height of the ruminal contents (ventral rumen). Prior to sampling, ruminal pH was measured by direct insertion into the rumen of a pH probe connected to a model 340i portable pH meter (WTW, Weinheim, Germany) subjected to a 2-point calibration (pH 4.01 and 7.00) just prior to use. At the time of collection, ruminal digesta were tightly hand-squeezed through 4 layers of cheesecloth, to separate the solid (SO) and liquid (L) phases. Volatile fatty acids (VFA) were determined from the L phase of ruminal digesta by HPLC (Weimer et al., 1991).

### Microbial DNA isolation

Microbial DNA was extracted from the L phase directly, but the SO phase was first homogenized in a blender with chilled extraction buffer (100 m*M* Tris/HCl, 10 m*M* EDTA, 0.15 *M* NaCl pH 8.0) to release the solids-associated bacteria as described by Stevenson and Weimer (2007). In brief, DNA was extracted from 25 mL of L phase and 25 g of SO phase of ruminal digesta using a series of wash steps with the extraction buffer followed by lysis of the microbial cells in a bead-beater, extraction with combinations of phenol/chloroform and precipitation with isopropanol (Stevenson and Weimer, 2007). The method is essentially identical to the PCSA method of Henderson et al. (2013) that has been shown to give very high yields of DNA that is likely to be most representative of the ruminal microbial community. The DNA obtained was resuspended in TE (10 m*M* Tris HCl, 1 m*M* EDTA, pH 8.0) and purified using an Electro-Eluter (C.B.S Scientific Company, Del Mar, CA).

### ARISA

The internally transcribed region (ITS) between the bacterial 16S and 23S rRNA genes was amplified using domain-specific bacterial primers ITSF (5′-GTCGTAACAAGGTAGCCGTA-3′) and ITSReub (5′-GCCAAGGCATCCAAC-3′). The primer ends were complementary to the respective positions 1423 and 1443 of the 23S rRNA and positions 38 and 23 of the 16S rRNA of *Escherichia coli* (Cardinale et al., 2004). The PCR components and the cycling conditions have been described previously (Weimer et al., 2010b). The PCR product obtained was resolved in a Beckman Coulter CEQ8000 Genetic Analysis System using the run parameters as described by Weimer et al. (2010b). In brief, 0.5 μL of the PCR product was mixed with 1 μL of Beckman Coulter WelRed #1 infrared fluorescent dye-labeled DNA standard ladder (MapMarker 1000, Bio Ventures, Murfreesboro, TN) and 39 μL of sample loading solution (Beckman Coulter) and loaded into microtiter plates. Capillary electrophoresis was conducted following the manufacturer's instructions after covering the liquid surface of the wells with molecular biology grade mineral oil.

The raw data obtained from capillary electrophoresis were imported into GeneMarker software (v. 1.75, Soft Genetics LLC, State College, PA) for further analysis. Peak detection and quantification was performed using the settings for amplicon fragment length polymorphism analysis described in the GeneMarker manual. Peak sizes (bp) were determined using the DNA standard ladder described above. Baseline subtraction and peak smoothing was performed as described in the GeneMarker manual. The panel generated by the software was screened manually to remove any questionable peaks caused by pull-up from the dyed DNA standard. All peaks corresponding to AL > 112 bp were exported for correspondence analysis (described under the Statistical Analysis Section).

### Library preparation and 454 pyrosequencing

Based on similarities in BCC of the 3 consecutive daily samplings within heifer X period, as revealed by ARISA (see Results), DNA samples from each separate ruminal fraction (L or SO) from the 3 consecutive daily samplings from each heifer were pooled in equimolar proportions, resulting in a total of 30 samples. L and SO samples corresponding to one sampling day during period one from heifer 3292 were not included in the analysis as this sampling was deemed to be an outlier (see Statistical Analyses, below). Amplification was achieved using custom designed primers containing the Roche 454 A or B Titanium sequencing adaptors on the forward primer, 926F-5′-CCTATCCCCTGTGTGCCTTGGCAGTCTCAGAAACTYAAAKGAATTGACGG -3′, and one of 30 unique barcodes, 5 bp in length, as indicated by XXXXX in the reverse primer, 1392R-5′-CCATCTCATCCCTGCGTGTCTCCGACTCAG - XXXXX-ACGGGCGGTGTGTRC-3′. Five 20 μl PCR reactions, which included 10 ng of template DNA, 0.5 μM of each primer, and 18 μL high-fidelity DNA polymerase Platinum Blue master mix (Invitrogen by Life Technologies, Grand Island, NY) were performed for each sample. PCR conditions consisted of an initial denaturation at 94°C for 2 min followed by 30 cycles of 94°C for 30 s, 50°C for 45 s, and 68°C for 105 s, with a final extension at 68°C for 10 min. Products from the five reactions were pooled, purified, and concentrated to 50 μL using the PureLink™ Quick PCR Purification Kit (Invitrogen by Life Technologies). One last purification step was performed by excising the appropriately sized amplicon (~550 bp) from a 1% low-melt agarose gel after electrophoresis, followed by recovery using the Zymoclean™ Gel DNA Recovery Kit (Zymo Research Corp., Irvine, CA). Each sample was quantified using a Qubit® Fluorometer (Invitrogen, San Diego, CA), and pooled at an equimolar ratio to create a single library at 1 × 10^9^ molecules per μL. The library was subsequently diluted to a concentration of 1 × 10^6^ molecules per μL and used as template for emPCR at a ratio of 0.8 molecules per DNA capture bead. Bead recovery and sequencing were performed following the manufacturer's guidelines for a Roche 454 GS Junior pyrosequencer with the Lib-L kit and Titanium chemistry (Roche Applied Science, Indianapolis, IN).

### qPCR primers

The primers used for qPCR have been described previously (Stevenson and Weimer, 2007) and their currently known specificities were determined by *in silico* re-analysis in Genbank. The genus *Prevotella* forward primer (PreGen4F 5′-GGT TCT GAG AGG AAG GTC CCC-3′) matched over 100 *Prevotella* sequences plus 11 species representing other genera, but no *Alloprevotella*; the reverse primer (PreGen4R 5′-TCC TGC ACG CTA CTT GGC TG-3′) matched only *Prevotella* sequences and one *Alloprevotella* sequence, but none of the 11 non-*Prevotella* sequences that matched the forward primer. The *M. elsdenii* forward primer (MegEls2F 5′-AGA TGG GGA CAA CAG CTG GA-3′) matched only this species and one *Anaeroglobus* species, but the MegEls2R 5′-CGA AAG CTC CGA AGA GCC T-3′) primer matched only *M. elsdenii*. The *B. fibrisolvens* primers displayed less specificity, reflecting the considerable taxonomic uncertainty of *B. fibrisolvens* (Willems and Collins, 2009). Among strains identified at the family level or below, and excluding taxonomically unassigned sequences, the forward primer (ButFib2F 5′-ACC GCA TAA GCG CAC GGA-3′) matched 8 non-*Butyrivibrio* species within the Lachnospiraceae and Clostridiaceae, while the reverse primer (ButFib2R 5′-CGG GTC CAT CTT GTA CCG ATA AAT-3′) matched 7 strains of *Pseudobutyrivibrio*. However, only one non-*Butyrivibrio* strain, *Pseudobutyrivibrio ruminis* Ce1, matched both the forward and reverse primers. Consequently we refer to the assemblage identified by the *Butyrivibrio* primers as the “*Butyrivibrio fibrisolvens* group” to indicate that the primers, though representative of a large number of *B. fibrisolvens* strains, were not completely monospecific.

### qPCR

Quantitative real time (qRT)-PCR assays for specific bacterial taxa were conducted with the L phase of ruminal digesta, using the primers described above. The standards for *M. elsdenii, B. fibrisolvens* group, and genus *Prevotella* assay were prepared by making a serial dilution of the genomic DNA from a pure culture of the strains T81, H17c, and *P. brevis* GA33, respectively. The standards and the set of samples belonging to each period were run in the same plate in triplicate. The relative population size (RPS) of the target bacterium was determined as the ratio of the amplification of target taxon 16S rRNA copy numbers to the amplification of the background obtained by amplifying the 16S rRNA gene with bacterial primers (BAC338F and BAC805R, Yu et al., 2005). The qRT-PCR was conducted using POWER SYBR Green PCR Master Mix (Applied Biosystems, Warrington, UK), forward and reverse primers (25 pmol of each primer/reaction) and approximately 20 ng of template DNA in a final volume of 25 μL per reaction. Quantitative real time-PCR assays were conducted using Applied Biosystems Prism 7300 sequence detection system. The amplification conditions were 40 cycles of 95°C for 15 s and an annealing and extension period of 60 s (at 59°C for *M. elsdenii*, 60°C for *B. fibrisolvens*, and 60°C for *Prevotella*). The efficiency of the PCR was calculated as the negative reciprocal of the slope of the line obtained by plotting Ct vs. log DNA concentrations of the standard dilution series.

### Statistical analysis

For ARISA, the data matrix resulting from the export of peak areas from GeneMarker was analyzed by correspondence analysis following the method of Ludwig and Reynolds (1988) using custom software written in the C programming language. One ordination point (out of 90 total) corresponding to the L phase of one heifer in the first period was identified as an outlier (geometric distance from the origin 6.4 times that of the next most distant point from the origin). This sample together with the corresponding sample in the SO phase was excluded from the analysis. The ordination points for the first 2 components of the correspondence analysis were plotted as scatter plots. The relative peak areas obtained from ARISA were analyzed for the shifts in BCC using analysis of similarity (ANOSIM; Clarke, 1993). Four data matrices were constructed from the ARISA data (L and SO phases for both OP and OH diets) corresponding to each heifer. Each data matrix contained 281 rows (corresponding to the different amplicon lengths, **AL**) and 9 columns (3 daily samples for each of the 3 periods).

For pyrosequencing, the sequence processing and data analysis was performed using the program mothur v.1.33.3 (Schloss et al., 2009) with default command parameters, unless specified. Flowgrams were trimmed to 450 flows and de-noised using the *shhh.flows* command, the mothur implementation of the AmpliconNoise algorithm (Quince et al., 2011). The resulting sequences were trimmed (pdiffs = 2, bdiffs = 0, maxhomop = 6, minlength = 250) followed by alignment against the SILVA 16S rRNA gene reference alignment database (Pruesse et al., 2007). Chimera detection was performed using the *chimera.uchime* command (http://drive5.com/uchime) on a screened version of the alignment (*filter.seqs*) that had been reduced using *unique.seqs* and *pre.cluster* (diffs = 2). The *cluster* command was used to assign sequences to operational taxonomic units (OTUs) using the nearest neighbor algorithm. All subsequent OTU-based analyses were performed using a cutoff of 0.03. Classification of OTUs was achieved using the Greengenes database (DeSantis et al., 2006) with a confidence level of at least 80 percent. Sequences classified as either domain Eukaryota or domain Archaea were removed from all subsequent analyses. Comparisons of taxa relative abundances among the 4 groups (OP/L, OP/SO, OH/L, OH/SO, encompassing the two forage types OH and OP and the two ruminal phases L and SO) were normalized by subsampling to 1856 sequences, the size of the smallest group. Diversity and coverage metrics were obtained using the command *summary.single* (calc = sobs-chao-ace-jack-bergerparker-shannon-simpson-coverage,label = 0.03, subsample = T). Analysis of community similarities (ANOSIM) (Clarke, 1993) was performed using the *anosim* command. The command *tree.shared* (calc = braycurtis,label = 0.03,subsample = T) using unweighted pair group method with arithmetic mean (UPGMA) clustering was performed to construct the dendrogram showing the relationships among the bacterial communities. Weighted UniFrac (Lozupone and Knight, 2005) was performed on the tree generated from the *clearcut* command using the command *unifrac.weighted* (subsample = T).

Ruminal fermentation variables and the RPS of *B. fibrisolvens* and *M. elsdenii* in L phase of ruminal digesta, as determined by qPCR, were averaged per heifer per period and analyzed using the MIXED procedure of SAS v.9.4 (SAS, Cary, NC). Heifer within diet was the experimental unit with diet and period designated as fixed effects in the model. Least square means (LSM) for diet and heifer within diet were determined and declared significant when *P* < 0.05. Differences in the LSM were determined using ADJUST = Tukey option in the LSM statement. Correlations between microbial taxa and the mol fraction or millimolar concentrations of individual VFA were determined using the CORR procedure in SAS.

### Accession numbers

Raw sequence files for each of the 30 samples analyzed by 454 pyrosequencing have been deposited at the National Center for Biotechnological Information (NCBI) Short Read Archive and can be found under project accession PRJNA253331.

## Results

### Nutrient composition of forages

Nutrient composition of the forages is presented on a dry matter basis in Table 1. Overall the OP averaged across periods had a higher WSC and CP, and lower NDF and ADF contents than did the OH. However, direct comparison of OP and OH harvested during period 2 revealed no difference in CP, NDF, ADF, ADL, or ash content (all *P* > 0.6); WSC was numerically higher in OH than in OP, but the difference was not significant (*P* = 0.16).

**Table 1:** **Nutrient compositions of orchardgrass used in this study^a^**.

	**Pasture**			**Hay^b^**
	**Period 1**	**Period 2**	**Period 3**	**Period 2**
Crude protein	11.8 ± 0.10	13.3 ± 0.08	23.5 ± 0.04	13.3 ± 0.12
Water-soluble carbohydrate	9.66 ± 2.95	4.54 ± 0.26	8.90 ± 1.00	6.28 ± 0.89
Neutral detergent fiber	55.3 ± 0.46	63.1 ± 0.01	50.7 ± 0.07	62.7 ± 0.57
Acid detergent fiber	28.8 ± 0.54	34.0 ± 0.52	25.0 ± 0.13	33.1 ± 0.25
Acid detergent lignin	3.63 ± 0.15	3.56 ± 0.64	3.04 ± 0.43	3.66 ± 0.06
Ash	2.16 ± 0.46	1.27 ± 0.64	1.48 ± 0.05	1.23 ± 0.59

a *Data are mean values ±SD from duplicate samples, expressed on a percent dry matter basis*.

b *Hay was prepared from orchardgrass grown for 21 days of growth, to the same growth stage (vegetative) that pasture was grazed. The hay was stored under cover until the OH stage began (day 24 of the pasture period)*.

### Ruminal VFA

**Figure 1:**
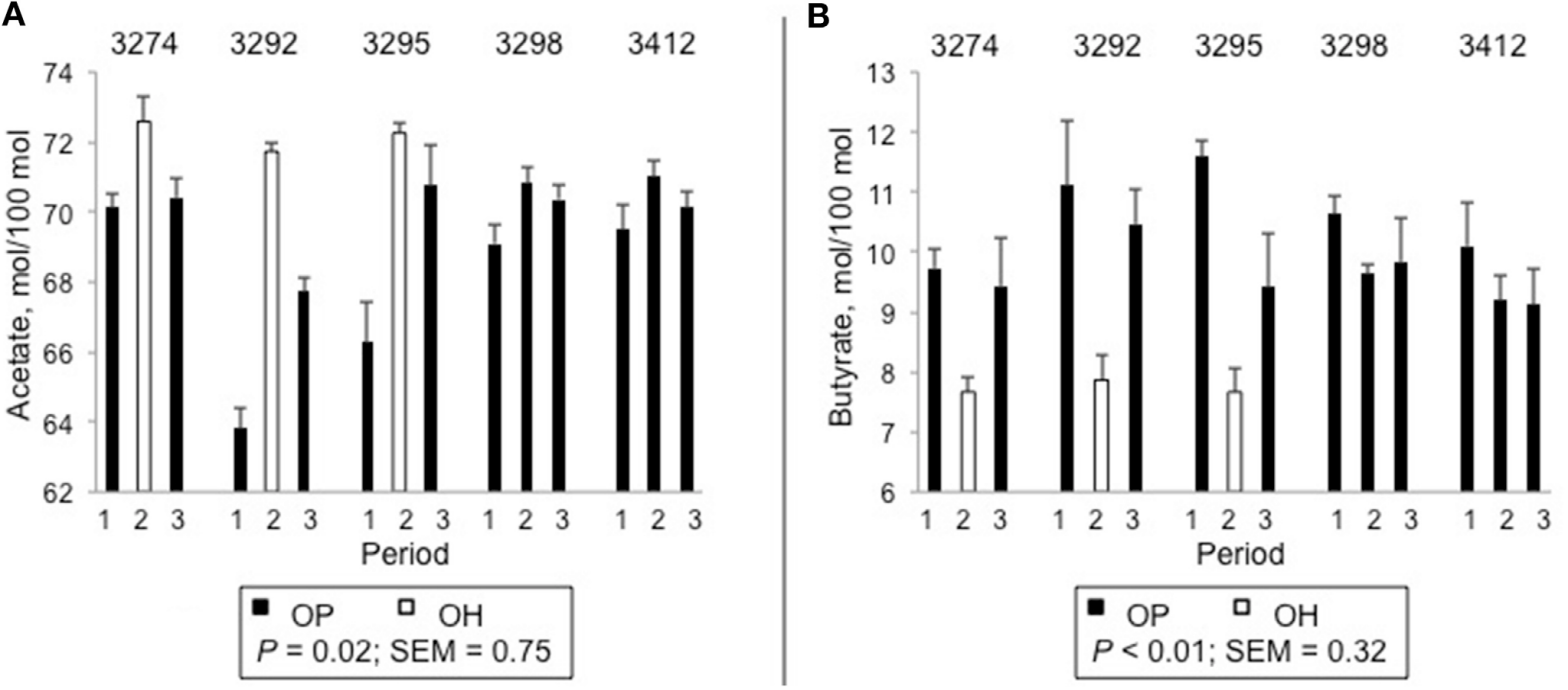
**Molar percentages of ruminal acetate (A) and butyrate (B) in individual heifers across periods**. Diets were orchardgrass pasture (OP) and orchardgrass hay (OH) harvested from at the vegetative stage of growth. Comparisons reflect differences between OP and OH in heifers that received both diets. s.e.m. = standard error of the mean.

Ruminal pH was not influenced by forage source (*P* = 0.40, Table 2). Total VFA concentration was greater for OH than for OP (*P* = 0.02). In addition, there were substantial differences in the molar proportions of the ruminal VFA. The proportion of acetate was greater for OH than for OP (*P* = 0.02) and the proportion of valerate tended to be greater for OH than OP (*P* = 0.05). The molar proportions of butyrate (*P* < 0.01), isobutyrate (*P* < 0.01) and the co-eluting pair isovalerate + 2-methylbutyrate (*P* < 0.01) were greater for OP than for OH. Variation in the molar proportions of acetate and butyrate in individual animals across the periods is plotted in Figure 1. All 3 heifers subjected to dietary change (3274, 3292, and 3295) displayed increased molar proportions of acetate (Figure 1A) and decreased proportions of butyrate (Figure 1B) upon switch from OP to OH, and a return to the original proportions upon a return to OP. By contrast, the two heifers maintained on OP (3298 and 3412) showed no changes in the proportions of these VFA across periods (Figures 1A,B).

**Table 2:** **Least-square means of ruminal fermentation variables in heifers grazing orchardgrass pasture (OP) or consuming orchardgrass hay (OH)^a^**.

**Variable^b^**	**Diet**			
	**OH**	**OP**	**s.e.m**.	***P*-value^d^**
Ruminal pH	6.35	6.26	0.07	0.40
Total VFA (m*M*)	182.2	132.7	11.1	0.02
VFA mol (100 mol)^−1^				
Acetate	72.2	68.9	0.75	0.02
Propionate	17.1	16.6	0.49	0.49
Butyrate	7.73	10.1	0.32	<0.01
Isobutyrate	0.78	1.35	0.07	<0.01
Valerate	1.29	1.11	0.05	0.05
Isovalerate + 2-methylbutyrate^c^	0.91	1.90	0.13	<0.01

a *Hay was harvested after 21 days of growth of orchardgrass at the same growth stage (vegetative) that pasture was grazed*.

b *VFA = volatile fatty acid*.

c *Co-eluted on HPLC analysis*.

d *Comparison across all heifers*.

### Bacterial community composition

Shifts in BCC across period were first examined using ARISA. The number of discrete amplicon lengths (AL) detected across all samples by the analysis of raw data generated from capillary electrophoresis of PCR products was 281, spanning a range of 61–798 bp. The mean number of AL detected in individual samples was 113 (minimum = 44, maximum = 159). The mean number of AL detected in the SO digesta were greater than L digesta (least-square means (LSM) of 118.3 for SO and 106.8 for L; S.E.D. = 3.47, *P* = 0.021). The mean number of amplicons did not differ between forage form for the SO digesta (LSM =120.0 for OH vs. 116.6 for OP; S.E.D. = 6.7, *P* = 0.614), or for the L digesta (LSM = 101.4 for OH and 112.2 for OP; S.E.D. = 7.47, *P* = 0.141). Correspondence analysis of the ARISA profile across the entire data set revealed that the first 2 components contributed 4.1% and 3.5%, respectively, to the total variation in the profile. These low percentages are consistent with the high dimensionality of the data (i.e., 113 different AL in a sample would provide 113 dimensions for analysis).

The ordination points from correspondence analysis of the ARISA profiles are shown Figure S1. For the 3 heifers (3274, 3292, and 3295) that were subjected to the OH treatment (i.e., the dietary sequence pasture/hay/pasture), the ordination points corresponding to the OH diet clustered separately from those on OP. It should be noted that the ordination points corresponding to OP in the third period (following transition from OH to OP) were close to those of OP in the first period (communities before changing to OH diet) and were generally well separated from those of OH, suggesting a return to a BCC similar to that present before the dietary switch. The ordination points corresponding to heifers 3298 and 3412, which remained on OP throughout the periods, were more closely clustered, indicating smaller changes in BCC over the course of the experiment. Analysis of similarity (ANOSIM) on the ARISA data revealed that the L and SO communities differed (*R* = 0.179, *P* = 0.005). Differences between OP and OH communities, taken as a whole, were confounded by the large differences in the L and SO communities. However, ANOSIM conducted within heifer and phase revealed that of OP vs. OH communities differed (Figure S1). For each heifer x period combination, the ordination points for the three successive ruminal sampling days within period were closely spaced, indicating that BCC had stabilized by the end of each period.

**Figure 2:**
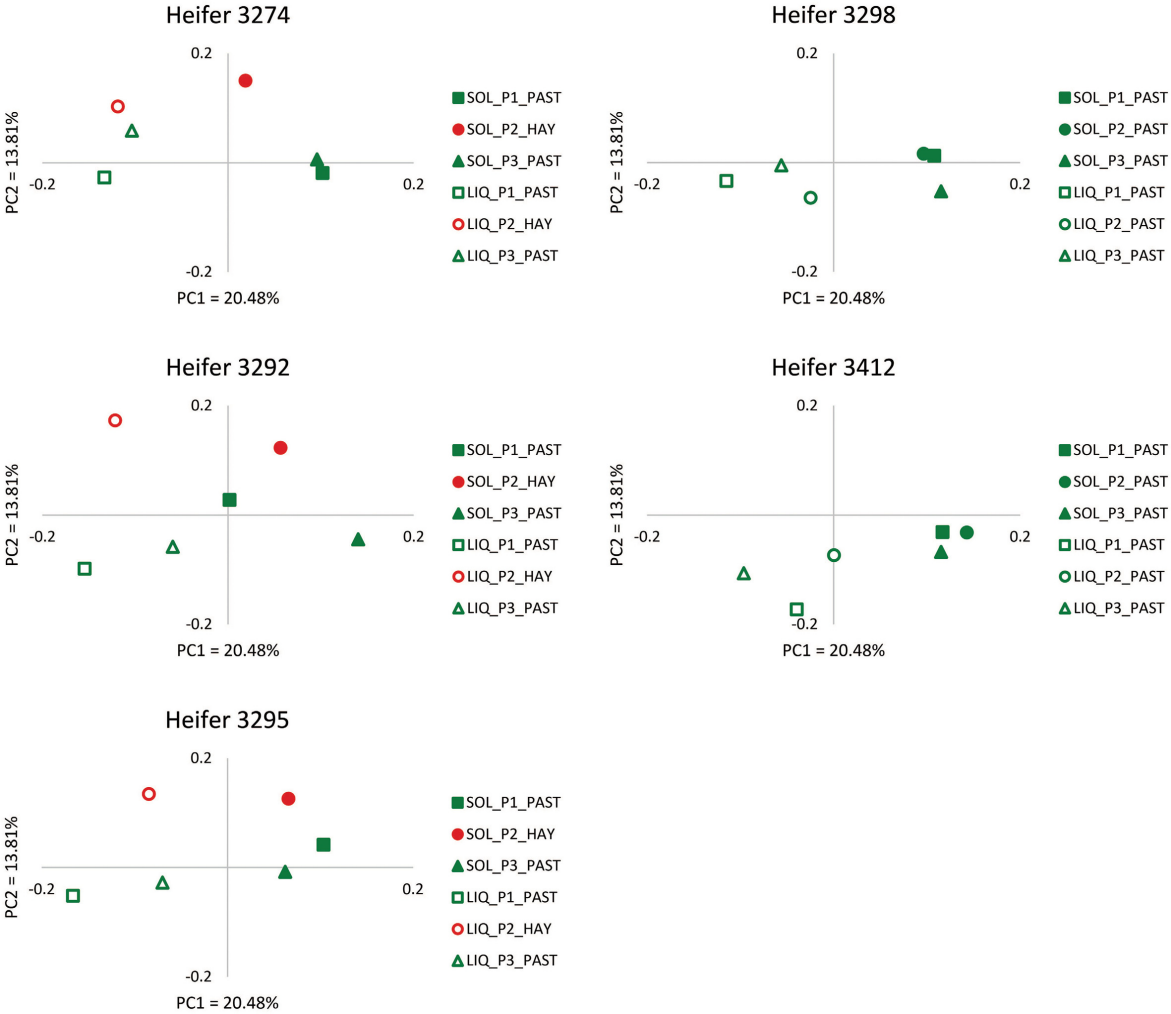
**Scatter plots of PC1 (x-axis) vs. PC2 (y-axis) from weighted UniFrac principal component analysis (PCoA) of the bacterial communities as determined by 454 pyrosequencing for each of the five heifers**. The amounts of variation explained by PC1 and PC2 are 20.48 and 13.81% respectively. Subsampling was performed to normalize among the samples.

**Figure 3:**
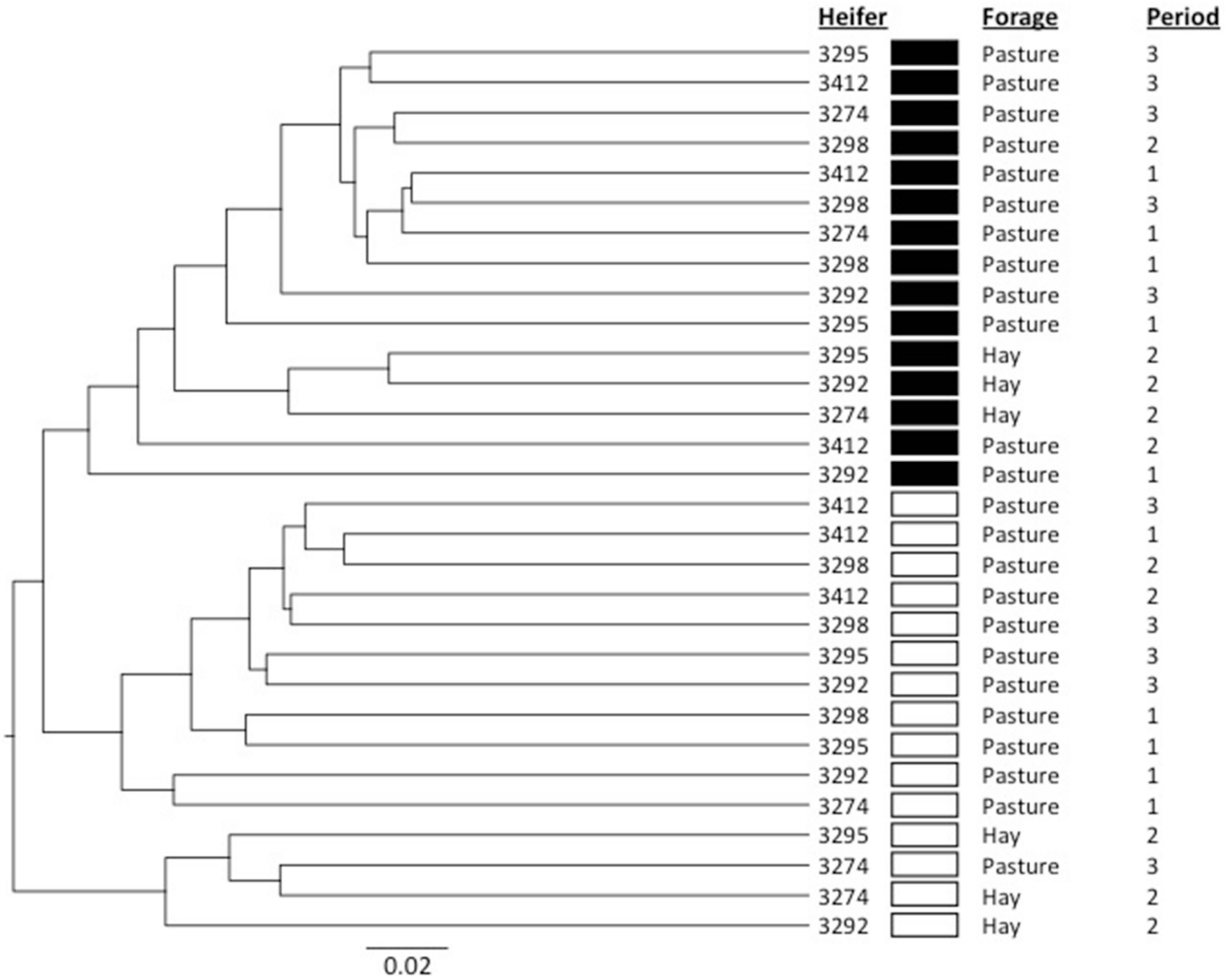
**Dendrogram showing the relationships among the bacterial communities as determined by 454 pyrosequencing**. The dendrogram was constructed using the Unweighted Pair Group Method with Arithmetic Mean (UPGMA) algorithm and Bray-Curtis calculation for determining the distance between communities. Filled boxes indicate samples from the solid phase of rumen contents while hollow boxes indicate samples from the rumen liquid. Subsampling was performed to normalize among the samples.

In order to provide more phylogenetic information on BCC, the isolated DNA was subjected to 16S rRNA gene sequencing using 454 pyrotag sequencing. Three successive daily samples within each heifer × period combination (which as noted above were highly similar from ARISA) were pooled to reduce sample number from 90 to 30. A total of 94,224 sequences were obtained, with an average per pooled sample of 3141 sequences. The pooled samples contained an average of 233 unique OTUs (range 169–305), and Good's coverage values (Good, 1953) of ≥0.92 (Table S1). The shifts in BCC revealed by ARISA were generally confirmed by pyrotag sequencing data. In principal component analysis, linear distances between points representing each feeding period were smaller for each heifer on the OP treatment than on the OH treatment (Figure 2). Moreover, within each of the 3 heifers subjected to dietary shift, the linear distance between the points corresponding to the two OP periods were much smaller than the distances between the points corresponding to OP vs. OH, suggesting a return to a similar BCC following a return to OP. Bray-Curtis analysis (Figure 3) revealed that individual communities displayed strong groupings primarily by rumen digesta phase (SO vs. L), and secondarily by forage source (OP vs. OH). ANOSIM (Table S2) confirmed that the communities associated with the L and SO fractions were dissimilar (*P* < 0.001), as were the OP and OH communities (*P* < 0.02). In addition, an OTU-based analysis of the pyrosequencing data, using average-neighbor (cutoff = 0.03) rather than nearest-neighbor clustering, was performed for comparison, with effects similar to those above (Table S2).

**Figure 4:**
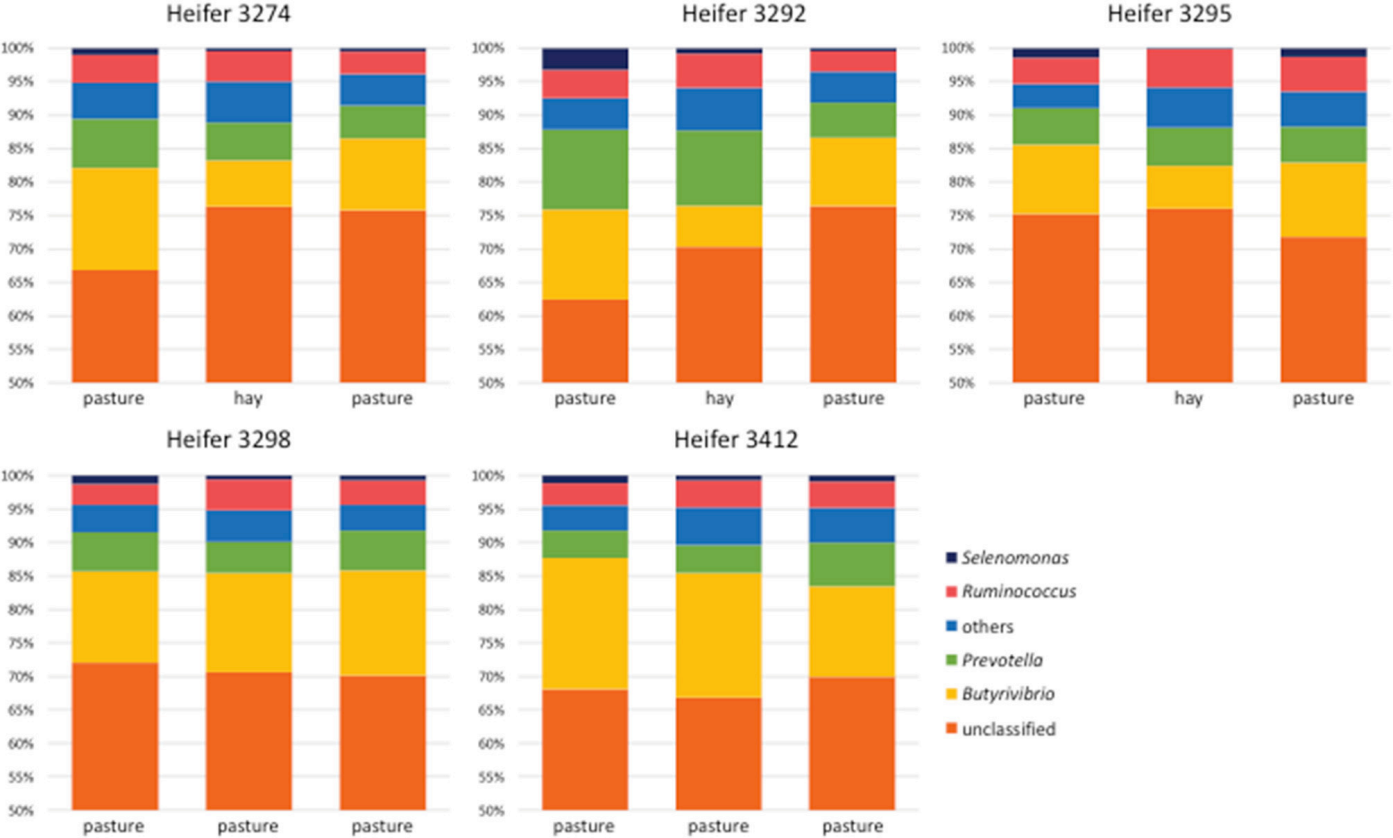
**Relative abundance of bacterial genera observed in all heifers during all sampling periods and representing on average ≥1% of the total 454 pyrosequencing reads recovered from each animal**. OTUs were classified to the genus level using the Greengenes database (DeSantis et al., 2006) with a consensus confidence threshold of 80 percent. OTUs from the liquid and solid phases from each heifer for each period were pooled to simplify the comparison. OTUs classified as genera that were not present in all five heifers during each sampling period and that were on average ≤1% relative abundance were condensed and are represented as “others.” Subsampling was performed to normalize among the samples. Regardless of heifer, or diet, most OTUs were unclassifiable at the genus level. The most dominant classifiable genera observed were *Butyrivibrio, Prevotella*, and *Ruminococcus*.

At the phylum level, the bacterial community was dominated by members of the phylum Firmicutes, which accounted for 58–86% of the sequences in individually sequenced samples (Figure S2 and Table S3). At the genus level, *Butyrivibrio* was the most abundant, accounting for up to 20% of the sequences, while *Prevotella* accounted for 2–14% and *Ruminococcus* for 2–7% (Figure 4 and Table S4). An unexpectedly high number (58–71%) of the sequences did not correspond to classified genera. At the genus level, only *Butyrivibrio* displayed significant diet-dependent shifts (Figure 4 and Table S4). In all 3 heifers receiving the OP-OH-OP treatment series, *Butyrivibrio* abundance decreased upon a shift from pasture to hay, and rebounded when the heifers were returned to pasture; by contrast, *Butyrivibrio* abundance in the two heifers retained on pasture did not show directional shifts across periods. Across animals, the least-square means for RPS of *Butyrivibrio* (expressed as percentage of total genus-level pyrotag sequencing reads) was higher on OP than on OH [13.8 vs. 8.3%, standard error of the difference (SED) = 1.2%, *P* = 0.0002], in accord with the higher molar proportion of butyrate on OP (Table 2 and Figure 1B). Linear regression of the RPS of genus *Butyrivibrio* and the molar proportion of butyrate in VFA revealed a positive correlation (*R* = +0.591 and *P* = 0.020 for liquid phase RPS; *R* = +0.285, *P* = 0.304 for solid phase RPS). By contrast, linear regression of the RPS of genus *Butyrivibrio* and the millimolar concentrations of butyrate did not display a significant positive correlation (*R* = −0.116, *P* = 0.680 for liquid phase RPS, *R* = −0.567, *P* = 0.027 for solid phase RPS). This is likely due to the poor ability of millimolar VFA concentrations to describe the ruminal fermentation and its nutritional consequences, as VFA concentrations are confounded by strong differences in ruminal volume, rate of passage and VFA uptake through the ruminal wall (Hall et al., 2014).

The observation that the bacterial community was dominated by Firmicutes, with smaller percentages of phylum Bacteroidetes and genus *Prevotella*, led us to quantify this genus using qRT-PCR. Both SO and L phase samples were examined, as *Prevotella* are known to utilize both soluble substrates and insoluble fiber and protein components of the diet. The RPS of *Prevotella*, expressed as a percentage of 16S rRNA gene copy number detected with a genus-level primer, ranged from 16 to 34 across samples, higher than those suggested by the pyrotag sequencing. The RPS of *Prevotella* was significantly higher in the SO phase (*P* = 0.03) and showed a tendency (*P* = 0.10) to be different among individual heifers, but did not differ between OP and OH (Table 3). The RPS of two known butyrate producing taxa, the *B. fibrisolvens* group and *M. elsdenii*, was analyzed in L phase of rumen digesta by qPCR. For both species RPS was very low (<0.1 and <0.01%, respectively; Table 3). The RPS of *M. elsdenii*, but not the *B. fibrisolvens* group, varied among heifers, but the RPS of both taxa did not vary between the diets.

**Table 3:** **Percentages of target species in rumen liquid samples relative to total eubacterial count determined by quantitative real-time PCR analysis in heifers grazing orchargrass pasture (OP) or consuming orchardgrass hay (OH) diets^a^**.

**Species1**		**Diet**				**Heifer × Diet**									
	**Phase**	**Hay**	**Pasture**	**s.e.m**.	***P*-value**	**3274**		**3292**		**3295**		**3298**	**3412**	**s.e.m**.	***P*-value**
						**Hay**	**Pasture**	**Hay**	**Pasture**	**Hay**	**Pasture**	**Pasture**	**Pasture**		
*M. elsdenii*	Liquid	0.001	0.003	0.001	0.21	0.001	0.001	0.001	0.004	0.001	0.002	0.003	0.007	0.002	0.39
*B. fibrisolvens*	Liquid	0.081	0.060	0.017	0.36	0.084	0.041	0.098	0.067	0.057	0.053	0.059	0.054	0.031	0.97
*Prevotella*	Solid	25.0	24.7	2.39	0.94	18.0	24.0	29.0	32.0	28.0	21.5	21.0	25.0	4.69	0.35
*Prevotella*	Liquid	33.7	23.9	3.07	0.07	17.0b	30.5b	68.0a	26.0b	16.0b	23.0b	19.7b	20.7b	5.53	0.01

a *The relative population size was determined as the ratio of copies of the 16S rRNA gene of the target species to copies of the 16S rRNA gene amplified with eubacterial primers and expressed as percentage*.

Analysis of community diversity and community richness (Table S5) revealed that community richness was not related (*P* > 0.05) to dietary sequence, phase of ruminal contents (SO vs. L), or the diets (OH vs. OP). The Shannon index of community diversity was significantly higher in the L phase than the SO phase (*P* < 0.001), and in the OH vs. OP community (*P* = 0.004), but the Simpson index did not differ (*P* > 0.05) within these comparison groups.

## Discussion

### Bacterial community composition

The shifts in BCC between OP and OH (evident from Figures 2, 3) occurred despite apparent similarities in their nutrient compositions (Table 1, cf. OH and OP during period 2). Although nutrient compositions were grossly similar, there may have been subtle differences between the two forms of forage with respect to physiological responses to the stresses induced by cutting and following ingestion into the rumen that may affect their susceptibility to enzymatic and microbial digestion, particularly of plant protein (“accelerated induced stress,” Kingston-Smith et al., 2008). The separation in the ordination points representing the L and SO phases of rumen digesta (Figure 3) was not as evident as that observed in our previous studies (Welkie et al., 2010; Weimer et al., 2010b; Mohammed et al., 2012). Those studies differed from the current study in that the diets offered were TMR-based diets that contained two forage sources (alfalfa and corn silages) plus grains, and several additional proteins sources, while the diets in this study were exclusively forage diets (in this case, nearly pure stands of orchardgrass). It is worth exploring the possibility that the relative proportion of SO- and L-associated bacteria could differ quantitatively between TMR and forage-only diets.

The pyrosequencing data revealed that, for all five heifers, bacteria in the phylum Firmicutes were more abundant in the rumen than were those of the Prevotellaceae (Figure S2). Previous studies employing the same primers and qPCR conditions have indicated that, in lactating cows fed a TMR containing alfalfa and corn silages, corn grain and protein concentrates, genus *Prevotella* represented 40–60% of the 16S rRNA gene copy number, as determined by qPCR (Stevenson and Weimer, 2007; Weimer et al., 2008). However, in this study, heifers fed all-forage diets (either as pasture or hay), genus *Prevotella* represented 16–34%, as determined by qPCR (Table 3), and <20%, as determined by pyrotag sequencing (Figure 4), of the 16S rRNA gene copy number.

Although there is an abundant literature on the effect of forage:concentrate ratio on the ruminal microbiome, only a few studies have examined the bacterial community in cattle on all-forage diets. Comparison of studies that used the 454 sequencing platform revealed substantial differences in ruminal community composition. For example, de Menezes et al. (2011) observed slightly higher proportions of Firmicutes than of Bacteroidetes in ruminally fistulated, lactating cows on cool-season C_3_ (ryegrass) pastures, while we observed somewhat higher ratios of Firmicutes to Bacterioidetes. McCann et al. (2014) examined the bacterial community in Brahman bulls from single samples collected after 60 d of grazing the warm season (C_4_) grass, Coastal bermudagrass (*Cyanodon dactylon* L). They reported that Bacteroidetes and Firmicutes represented 67.5 and 22.9% of the sequences at the phylum level. The substantial abundance of the Bacteroidetes could have been due to a combination of breed, forage type, collection of samples via stomach tube (which preferentially selects for planktonic cells), and the use of a commercial stool kit for DNA isolation (which results in low DNA yield and enrichment in Bacteroidetes; Henderson et al., 2013). Pitta et al. (2010) have previously demonstrated by 16S-based pyrotag sequencing that the abundance of genus *Prevotella* in crossbred beef steers varied with diet, and were substantially lower when the steers grazed bermudagrass than when they were switched to grazed winter wheat (24–33% vs. 36–56%). Further research is warranted to determine the relative importance of animal type and feeding regimen on the composition of the ruminal microbiome, preferably using the same community analysis techniques across experimental conditions.

### Ruminal VFA content

The molar proportions of individual VFAs in the rumen have potential to indicate shifts in BCC, because different ruminal microbes produce different VFA products (Russell, 2002). In the present study, a greater proportion of butyrate and a lesser proportion of acetate were observed from OP than from OH when averaged across OP periods (Table 2). Although it is tempting to ascribe these differences to compositional differences (e.g., NDF and ADF contents of OP averaged across periods was higher than those of the OH used in period 2), the same trends in acetate and butyrate were observed within period 2, in which OH and OP did not differ in composition (Table 1).

Differences in VFA proportions, as well as in total ruminal VFA concentration (higher for OH than for OP) may have been related to differences in the availability of specific nutrients between OP and OH, or to subtle differences in animal physiology and behavior (intake, rate of passage, and meal patterning) when consuming OP vs. OH, which were not measured in this study. Although its source remains to be identified, the greater proportion of butyrate for OP than OH reflects higher RPS of the abundant genus *Butyrivibrio* (Figure 4 and Table S4), which is regarded as a major ruminal butyrate producer (Miller and Jenesel, 1979). Another ruminal bacterium, *M. elsdenii*, is also known to produce large amounts of butyrate (Rogosa, 1971), but its relative abundance in this study was very low, and did not differ between OH and OP (Table 3). It is also possible that other ruminal microbes may be involved. The major butyrate producers in the rumen include several species of protozoa (Heald and Oxford, 1953; Abou Akkada and Howard, 1960; Yarlett et al., 1985), and Holden et al. (1994) reported that cows grazing primarily orchardgrass had three times higher protozoal counts than cows fed hay or silage. Huws et al. (2009), in a study comparing fresh perennial ryegrass with grass conserved as hay, found greater WSC and greater total protozoal density for fresh grass than for hay. Based on the above discussion, we speculate that the greater proportion of butyrate for OP than OH could be due to a greater population sizes of genus *Butyrivibrio* (Figure 4) and possibly protozoa (not tested in this study), but a connection to WSC content could not be established because WSC content did not differ between OH and OP when both were fed over the same time period (Table 1).

The branched-chain isomers of butyric and valeric acids (collectively termed branch-chain VFA, or **BCVFA**) are protein degradation products resulting from the deamination of amino acids in the rumen (Russell, 2002). Greater proportions of isobutyrate and of the chromatographically co-eluting isovalerate plus 2-methylbutyrate in the rumen for OP than OH are also consistent with the greater RPS of *Butyrivibrio*, which are also known to hydrolyze protein and ferment amino acids to ammonia and VFA (Willems and Collins, 2009), presumably including the BCVFA.

## Conclusions

This study demonstrated that the small number of heifers tested displayed a statistically significant shift in BCC as they were switched from OP to OH, with a decrease in the relative population size of the abundant genus *Butyrivibrio* on OH being particularly noteworthy. This shift was accompanied by, and consistent with, differences in the relative proportion of ruminal VFA (increased acetate and decreased butyrate and branched-chain VFA for OH relative to OP). For both OP and OH treatments, the ruminal abundance of genus *Prevotella* was strikingly lower than has been widely observed in cows fed a TMR of forages, grain and protein concentrates.

## Author contributions

Conceived and designed the experiments: Geoffrey E. Brink, Paul J. Weimer, Riazuddin Mohammed, Karen A. Beauchemin. Performed the experiments: Riazuddin Mohammed, Geoffrey E. Brink, David M. Stevenson, Anthony P. Neumann, Paul J. Weimer. Analyzed the data: Riazuddin Mohammed, David M. Stevenson, Geoffrey E. Brink, Anthony P. Neumann, Garret Suen, Paul J. Weimer. Contributed reagents/materials/analysis tools: Geoffrey E. Brink, Paul J. Weimer, David M. Stevenson, Garret Suen, Karen A. Beauchemin. Wrote the paper: Riazuddin Mohammed, Paul J. Weimer, Geoffrey E. Brink. All authors agree to be accountable for all aspects of the work.

### Conflict of interest statement

The authors declare that the research was conducted in the absence of any commercial or financial relationships that could be construed as a potential conflict of interest.
