# Supplementary material for: Bacterial communities in the rumen of Holstein heifers differ when fed orchardgrass as pasture vs. hay
Source: Front Microbiol. 2014 Dec 9;5:689. doi: 10.3389/fmicb.2014.00689 (PMC4260508; doi:10.3389/fmicb.2014.00689)
Supplement: Supplementary file 1 [file Table1.DOCX]

**SUPPLEMENTARY DATA for Mohammed et al.**

**Table S1.** Sequence distribution and coverage for samples analyzed by 454 pyrosequencing

| Heifer | Rumen Fraction | Period | Diet | Number of Sequences^α^ | Good’s Coverage^β^ | OTUs per sample^β^ |
| --- | --- | --- | --- | --- | --- | --- |
| 3274 | Solid | 1 | OP | 4232 | 0.942 | 218.49 |
| 3274 | Liquid | 1 | OP | 2992 | 0.936 | 257.61 |
| 3274 | Solid | 2 | OH | 4353 | 0.933 | 270.48 |
| 3274 | Liquid | 2 | OH | 2531 | 0.921 | 304.47 |
| 3274 | Solid | 3 | OP | 4078 | 0.938 | 234.89 |
| 3274 | Liquid | 3 | OP | 3887 | 0.920 | 290.38 |
| 3292 | Solid | 1 | OP | 3249 | 0.951 | 195.41 |
| 3292 | Liquid | 1 | OP | 3850 | 0.953 | 179.03 |
| 3292 | Solid | 2 | OH | 3328 | 0.942 | 223.22 |
| 3292 | Liquid | 2 | OH | 3773 | 0.928 | 272.78 |
| 3292 | Solid | 3 | OP | 2443 | 0.954 | 178.39 |
| 3292 | Liquid | 3 | OP | 3441 | 0.949 | 202.44 |
| 3295 | Solid | 1 | OP | 2826 | 0.959 | 168.69 |
| 3295 | Liquid | 1 | OP | 2466 | 0.933 | 253.97 |
| 3295 | Solid | 2 | OH | 2493 | 0.930 | 245.57 |
| 3295 | Liquid | 2 | OH | 1856 | 0.936 | 270.00 |
| 3295 | Solid | 3 | OP | 3851 | 0.939 | 219.96 |
| 3295 | Liquid | 3 | OP | 1870 | 0.945 | 234.25 |
| 3298 | Solid | 1 | OP | 4255 | 0.947 | 210.96 |
| 3298 | Liquid | 1 | OP | 2510 | 0.928 | 271.81 |
| 3298 | Solid | 2 | OP | 4221 | 0.935 | 239.53 |
| 3298 | Liquid | 2 | OP | 2433 | 0.931 | 248.03 |
| 3298 | Solid | 3 | OP | 1966 | 0.942 | 218.77 |
| 3298 | Liquid | 3 | OP | 2750 | 0.925 | 277.57 |
| 3412 | Solid | 1 | OP | 4975 | 0.946 | 205.17 |
| 3412 | Liquid | 1 | OP | 2189 | 0.947 | 204.44 |
| 3412 | Solid | 2 | OP | 3870 | 0.938 | 218.28 |
| 3412 | Liquid | 2 | OP | 2742 | 0.939 | 224.93 |
| 3412 | Solid | 3 | OP | 2456 | 0.942 | 215.87 |
| 3412 | Liquid | 3 | OP | 2338 | 0.938 | 230.33 |

^α^ After processing using mother (see materials & methods), avg. sequence length = 237 bp

^β^ Average of 1000 iterations of subsampling to 1856 sequences

**Table S2.** Analysis of similarities (ANOSIM) between bacterial communities from liquid (L) and solid (SO) ruminal fractions as well as orchardgrass pasture (OP) and orchardgrass hay (OH)

| Comparison | Method | Similarity Calculator | # of Iterations | R | *P*-value |
| --- | --- | --- | --- | --- | --- |
| L vs S | Nearest neighbor | Bray-Curtis | 1000 | 0.5546 | < 0.001 |
| L vs S | Nearest neighbor | Yue & Clayton theta | 1000 | 0.3186 | < 0.001 |
| L vs S | Average neighbor | Bray-Curtis | 1000 | 0.5470 | < 0.001 |
| L vs S | Average neighbor | Yue & Clayton theta | 1000 | 0.2966 | < 0.001 |
| OP vs OH | Nearest neighbor | Bray-Curtis | 10000 | 0.3896 | 0.0046 |
| OP vs OH | Nearest neighbor | Yue & Clayton theta | 10000 | 0.3384 | 0.0124 |
| OP vs OH | Average neighbor | Bray-Curtis | 10000 | 0.4296 | 0.0012 |
| OP vs OH | Average neighbor | Yue & Clayton theta | 10000 | 0.3864 | 0.0018 |

^a^ Bacterial Communities as determined by 454 pyrosequencing of the V6-V8 16S rRNA gene. Subsampling of each of the 30 samples was performed to normalize among the communities

^b^ A higher R-value indicates greater differences in the BCC and the probability values represent the fraction of these R-values that exceeded the R-value obtained from the nonrandomized comparison.

**Table S3.** Percent relative abundance of phyla observed in ruminal solids and liquids from heifers consuming orchardgrass pasture (OP) or hay (OH) during the three experimental periods, determined by pyrosequencing analysis.^a^

| **Phylum** | Solid | | | | | | | | | | | | | | | Liquid | | | | | | | | | | | | | | |
| --- | --- | --- | --- | --- | --- | --- | --- | --- | --- | --- | --- | --- | --- | --- | --- | --- | --- | --- | --- | --- | --- | --- | --- | --- | --- | --- | --- | --- | --- | --- |
|  | 3274 | | | 3292 | | | 3295 | | | 3298 | | | 3412 | | | 3274 | | | 3292 | | | 3295 | | | 3298 | | | 3412 | | |
|  | OP | OH | OP | OP | OH | OP | OP | OH | OP | OP | OP | OP | OP | OP | OP | OP | OH | OP | OP | OH | OP | OP | OH | OP | OP | OP | OP | OP | OP | OP |
| Acidobacteria | ND | ND | ND | ND | ND | ND | ND | ND | ND | ND | ND | ND | ND | ND | ND | ND | ND | ND | ND | ND | ND | 0.05 | ND | ND | ND | ND | ND | ND | ND | ND |
| Actinobacteria | 0.11 | ND | ND | ND | ND | ND | ND | ND | ND | 0.05 | 0.11 | ND | ND | 0.27 | 0.16 | 0.05 | 0.11 | ND | ND | 0.27 | ND | 0.16 | 0.11 | 0.16 | 0.05 | ND | ND | ND | ND | 0.05 |
| Armatimonadetes | 0.05 | 0.05 | 0.05 | ND | ND | 0.11 | 0.05 | ND | ND | ND | 0.11 | 0.05 | 0.05 | 0.05 | 0.05 | 0.05 | 0.11 | ND | ND | ND | 0.05 | 0.27 | 0.05 | 0.11 | 0.05 | ND | 0.05 | ND | 0.11 | 0.05 |
| Bacteroidetes | 7.76 | 8.62 | 5.23 | 11.53 | 9.97 | 5.55 | 1.99 | 7.33 | 10.18 | 6.30 | 8.19 | 6.03 | 6.63 | 5.23 | 9.00 | 9.11 | 7.65 | 9.54 | 14.39 | 17.40 | 7.70 | 13.90 | 6.95 | 3.61 | 9.64 | 3.66 | 9.75 | 2.75 | 5.17 | 6.84 |
| Chloroflexi | 0.05 | 0.05 | 0.05 | ND | 0.05 | ND | 0.05 | ND | 0.05 | ND | 0.05 | 0.05 | 0.05 | ND | ND | 0.05 | 0.22 | 0.11 | 0.05 | 0.05 | ND | 0.22 | 0.16 | 0.11 | 0.16 | 0.11 | ND | ND | 0.05 | 0.05 |
| Cyanobacteria | 0.05 | 0.81 | 0.16 | 0.48 | 0.48 | 0.27 | 0.65 | 0.43 | 0.22 | 0.43 | 0.43 | 0.43 | 0.27 | 0.81 | 0.22 | 3.18 | 3.23 | 3.02 | 0.86 | 3.29 | 3.29 | 2.21 | 6.79 | 2.96 | 4.04 | 3.56 | 3.61 | 2.10 | 4.53 | 2.32 |
| Elusimicrobia | 0.05 | 0.22 | ND | ND | 0.05 | ND | ND | 0.16 | ND | 0.11 | 0.11 | 0.05 | ND | 0.05 | ND | 0.05 | 0.16 | 0.11 | ND | 0.11 | ND | ND | 0.32 | 0.11 | ND | 0.05 | ND | 0.05 | 0.11 | ND |
| Fibrobacteres | 0.11 | 0.27 | 0.22 | 0.27 | ND | ND | ND | ND | 0.16 | 0.11 | 0.16 | ND | 0.05 | 0.16 | ND | ND | ND | 0.05 | ND | 0.54 | ND | 0.05 | ND | ND | 0.16 | ND | 0.27 | ND | ND | ND |
| Firmicutes | 80.60 | 70.74 | 82.60 | 80.44 | 74.95 | 85.83 | 76.72 | 73.06 | 80.06 | 81.25 | 77.96 | 84.16 | 84.59 | 84.32 | 82.60 | 67.94 | 64.06 | 65.25 | 74.57 | 58.03 | 76.08 | 69.56 | 65.68 | 74.89 | 70.64 | 78.88 | 71.34 | 83.78 | 76.08 | 76.99 |
| Lentisphaerae | ND | 0.16 | ND | ND | ND | 0.05 | ND | ND | 0.11 | ND | 0.05 | ND | ND | 0.05 | ND | 0.05 | 0.38 | ND | ND | 0.11 | 0.05 | 0.16 | ND | 0.11 | ND | ND | ND | 0.05 | 0.05 | ND |
| MVP-15 | ND | 0.05 | 0.11 | ND | ND | ND | ND | ND | ND | ND | ND | ND | ND | ND | ND | 0.16 | 0.05 | 0.11 | ND | ND | ND | ND | ND | ND | ND | ND | ND | ND | ND | ND |
| Planctomycetes | 0.16 | 0.27 | 0.16 | 0.16 | ND | ND | ND | 0.05 | 0.32 | ND | 0.05 | 0.22 | 0.22 | 0.05 | 0.27 | 0.22 | 0.48 | 0.16 | ND | 0.05 | 0.48 | 0.65 | 0.11 | 0.27 | 0.48 | 0.16 | 0.32 | 0.38 | 0.11 | 0.75 |
| Proteobacteria | 0.27 | 0.22 | 0.27 | 0.48 | 0.11 | 0.22 | ND | ND | 0.27 | 0.11 | 0.43 | 0.22 | 0.16 | 0.70 | 0.38 | 1.56 | 1.35 | 0.86 | 0.59 | 1.40 | 0.70 | 0.65 | 0.86 | 0.70 | 1.29 | 1.19 | 0.70 | 0.38 | 0.75 | 0.97 |
| Spirochaetes | 0.05 | 0.54 | 0.05 | 0.16 | 0.54 | 0.05 | 0.16 | 0.32 | ND | 0.16 | 0.16 | 0.11 | 0.32 | 0.48 | 0.11 | 0.22 | 0.43 | 0.11 | 0.16 | 0.54 | 0.16 | 0.16 | 0.05 | 0.05 | 0.16 | 0.22 | 0.22 | 0.05 | 0.16 | 0.27 |
| Synergistetes | ND | 0.11 | ND | 0.11 | 0.05 | 0.05 | ND | ND | ND | 0.05 | ND | 0.05 | 0.05 | 0.11 | ND | 0.16 | 0.16 | 0.11 | 0.05 | 0.11 | ND | 0.05 | ND | 0.27 | ND | 0.05 | 0.11 | 0.05 | 0.16 | 0.27 |
| Tenericutes | 6.73 | 11.37 | 5.87 | 3.77 | 9.70 | 5.23 | 17.83 | 12.77 | 4.42 | 8.24 | 6.57 | 4.69 | 4.31 | 4.63 | 3.88 | 9.21 | 12.28 | 12.55 | 4.74 | 12.28 | 7.65 | 5.50 | 10.78 | 10.78 | 5.77 | 6.03 | 6.63 | 2.96 | 7.76 | 6.14 |
| TM7 | 0.05 | 0.11 | 0.11 | ND | 0.05 | 0.22 | 0.05 | 0.22 | ND | 0.05 | 0.16 | ND | 0.05 | ND | ND | 0.11 | 0.05 | 0.16 | 0.27 | 0.05 | 0.38 | 0.22 | 0.43 | 0.11 | 0.54 | 0.22 | 0.11 | 0.32 | ND | 0.11 |
| Verrucomicrobia | 0.11 | 0.22 | 0.11 | 0.05 | ND | ND | ND | ND | 0.05 | 0.05 | ND | ND | 0.05 | ND | ND | ND | 0.11 | 0.11 | ND | 0.05 | ND | 0.05 | ND | ND | ND | ND | ND | ND | ND | ND |
| WPS-2 | ND | ND | ND | ND | ND | ND | ND | ND | 0.11 | 0.05 | ND | ND | ND | ND | 0.05 | 0.11 | ND | ND | ND | ND | ND | ND | 0.11 | 0.05 | 0.22 | ND | ND | 0.11 | 0.05 | 0.43 |
| unclassified | 3.83 | 6.20 | 5.01 | 2.53 | 4.04 | 2.42 | 2.48 | 5.66 | 4.04 | 3.02 | 5.44 | 3.93 | 3.18 | 3.07 | 3.29 | 7.76 | 9.16 | 7.76 | 4.31 | 5.71 | 3.45 | 6.14 | 7.60 | 5.71 | 6.79 | 5.87 | 6.90 | 7.00 | 4.90 | 4.74 |

^a^ Subsampling of each of the 30 samples was performed to normalize among the communities. ND = Not Detected

**Table S4.** Percent relative abundance of genera observed in ruminal solids and liquids from heifers consuming orchardgrass pasture (OP) or hay (OH) during the three experimental periods, determined by pyrosequencing analysis.

| Genus | Solid | | | | | | | | | | | | | | | Liquid | | | | | | | | | | | | | | |
| --- | --- | --- | --- | --- | --- | --- | --- | --- | --- | --- | --- | --- | --- | --- | --- | --- | --- | --- | --- | --- | --- | --- | --- | --- | --- | --- | --- | --- | --- | --- |
|  | 3274 | | | 3292 | | | 3295 | | | 3298 | | | 3412 | | | 3274 | | | 3292 | | | 3295 | | | 3298 | | | 3412 | | |
|  | OP | OH | OP | OP | OH | OP | OP | OH | OP | OP | OP | OP | OP | OP | OP | OP | OH | OP | OP | OH | OP | OP | OH | OP | OP | OP | OP | OP | OP | OP |
| *Acetivibrio* | 0.11 | 0.05 | ND | ND | ND | 0.05 | ND | 0.05 | ND | ND | 0.05 | ND | 0.11 | 0.16 | ND | 0.38 | 0.16 | ND | ND | 0.05 | ND | ND | 0.16 | ND | ND | 0.16 | ND | ND | 0.05 | ND |
| *Acinetobacter* | ND | ND | ND | ND | 0.05 | ND | ND | ND | ND | ND | ND | ND | ND | ND | 0.05 | ND | ND | 0.22 | ND | 0.05 | 0.05 | ND | 0.05 | ND | ND | 0.11 | 0.05 | ND | ND | 0.16 |
| *Adlercreutzia* | 0.05 | ND | ND | ND | ND | ND | ND | ND | ND | ND | ND | ND | ND | ND | ND | ND | ND | ND | ND | ND | ND | ND | ND | ND | ND | ND | ND | ND | ND | ND |
| *Anaerofustis* | ND | 0.05 | ND | 0.05 | ND | ND | ND | 0.05 | 0.05 | ND | 0.05 | ND | ND | ND | ND | 0.11 | ND | ND | ND | ND | ND | ND | ND | 0.05 | ND | 0.05 | ND | ND | ND | ND |
| *Anaeroplasma* | 0.54 | 0.81 | 0.22 | 0.32 | 0.32 | 0.11 | 0.70 | 0.81 | 0.05 | 0.05 | 0.70 | 0.32 | 0.11 | 0.48 | 0.16 | 0.75 | 1.13 | 0.43 | 0.86 | 1.35 | 0.65 | 0.27 | 1.35 | 0.59 | 0.32 | 0.54 | 0.38 | 0.16 | 1.40 | 0.48 |
| *Atopobium* | ND | ND | ND | ND | ND | ND | ND | ND | ND | 0.05 | ND | ND | ND | ND | ND | ND | ND | ND | ND | ND | ND | 0.05 | ND | ND | ND | ND | ND | ND | ND | ND |
| *Bacillus* | ND | ND | ND | ND | ND | ND | ND | ND | ND | ND | ND | ND | ND | ND | ND | ND | 0.11 | ND | ND | 0.65 | ND | ND | 0.16 | ND | ND | ND | ND | ND | ND | ND |
| *Bacteroides* | ND | 0.05 | 0.11 | ND | ND | ND | ND | ND | ND | ND | 0.05 | ND | 0.05 | ND | ND | ND | ND | 0.05 | ND | ND | ND | 0.05 | ND | ND | 0.05 | ND | ND | ND | ND | ND |
| *Brevibacterium* | ND | ND | ND | ND | ND | ND | ND | ND | ND | ND | ND | ND | ND | ND | ND | ND | ND | ND | ND | 0.16 | ND | ND | ND | ND | ND | ND | ND | ND | ND | ND |
| *Bulleidia* | 0.05 | ND | ND | ND | ND | 0.11 | ND | ND | ND | ND | 0.11 | 0.22 | ND | ND | ND | 0.11 | ND | 0.11 | 0.43 | ND | 0.16 | 0.11 | 0.05 | 0.27 | 0.11 | 0.11 | 0.05 | 0.11 | ND | 0.27 |
| *Butyrivibrio* | 17.35 | 8.41 | 13.09 | 9.86 | 8.19 | 11.91 | 11.75 | 8.62 | 12.45 | 14.12 | 14.06 | 18.91 | 20.64 | 21.50 | 15.73 | 13.15 | 5.39 | 8.41 | 17.08 | 4.09 | 8.51 | 8.94 | 4.15 | 9.86 | 13.15 | 15.63 | 12.50 | 18.64 | 15.73 | 11.37 |
| *Caulobacter* | ND | ND | ND | ND | ND | ND | ND | ND | ND | ND | ND | ND | ND | ND | ND | 0.05 | ND | ND | ND | ND | ND | ND | ND | ND | ND | ND | ND | ND | ND | ND |
| *Clostridium* | 0.11 | 0.43 | 0.38 | 0.22 | 0.05 | 0.11 | 0.32 | 0.38 | 0.16 | 0.27 | 0.27 | 0.16 | 0.16 | 0.16 | 0.22 | 0.32 | 0.11 | 0.32 | 0.05 | 0.32 | 0.16 | ND | 0.81 | 0.48 | 0.11 | 0.27 | 0.22 | 0.16 | 0.16 | 0.16 |
| *Coprococcus* | 1.94 | 1.13 | 0.81 | 2.42 | 1.08 | 0.59 | 0.86 | 0.75 | 0.43 | 0.75 | 0.38 | 0.65 | 0.54 | 0.92 | 0.81 | 1.08 | 0.65 | 0.48 | 0.48 | 1.29 | 0.86 | 0.86 | 1.08 | 0.48 | 0.65 | 0.75 | 0.43 | 0.38 | 0.86 | 0.86 |
| *Corynebacterium* | ND | ND | ND | ND | ND | ND | ND | ND | ND | ND | ND | ND | ND | ND | ND | ND | 0.05 | ND | ND | ND | ND | ND | ND | ND | ND | ND | ND | ND | ND | ND |
| *Dehalobacterium* | 0.05 | 0.43 | 0.32 | ND | 0.05 | ND | 0.05 | 0.11 | 0.22 | 0.16 | 0.16 | 0.11 | 0.16 | 0.11 | 0.43 | ND | 0.16 | 0.16 | ND | ND | ND | 0.05 | 0.16 | 0.16 | 0.05 | 0.16 | 0.16 | 0.05 | 0.11 | 0.16 |
| *Desulfobulbus* | ND | ND | ND | ND | ND | ND | ND | ND | ND | ND | ND | ND | ND | ND | ND | ND | ND | 0.05 | ND | ND | ND | ND | ND | ND | ND | ND | ND | ND | ND | ND |
| *Desulfovibrio* | ND | 0.05 | ND | 0.05 | ND | ND | ND | ND | 0.05 | ND | 0.11 | 0.16 | 0.05 | 0.11 | 0.11 | 0.11 | 0.05 | 0.05 | 0.11 | 0.11 | 0.05 | 0.11 | ND | ND | 0.27 | 0.11 | 0.22 | 0.11 | 0.05 | 0.05 |
| *Devosia* | ND | ND | ND | ND | ND | ND | ND | ND | ND | ND | 0.05 | ND | ND | ND | ND | ND | ND | ND | ND | ND | ND | ND | ND | ND | ND | ND | ND | ND | ND | ND |
| *Eubacterium* | 0.43 | 0.32 | 0.70 | 0.11 | 0.59 | 0.43 | 0.38 | 0.65 | 1.51 | 0.48 | 0.11 | 0.43 | 0.97 | 0.43 | 0.65 | 0.92 | 0.59 | 0.16 | 0.11 | 0.70 | 1.19 | 0.27 | 0.22 | 1.40 | 0.75 | 1.08 | 0.81 | 0.92 | 1.51 | 1.83 |
| *Exiguobacterium* | ND | ND | ND | ND | ND | ND | ND | ND | ND | ND | ND | ND | ND | 0.11 | ND | ND | ND | ND | ND | ND | ND | ND | ND | ND | ND | ND | ND | ND | ND | ND |
| *Fibrobacter* | 0.11 | 0.27 | 0.22 | 0.27 | ND | ND | ND | ND | 0.16 | 0.11 | 0.16 | ND | 0.05 | 0.16 | ND | ND | ND | 0.05 | ND | 0.54 | ND | 0.05 | ND | ND | 0.16 | ND | 0.27 | ND | ND | ND |
| *Hymenobacter* | ND | ND | ND | ND | ND | ND | ND | ND | ND | ND | ND | ND | ND | ND | ND | ND | ND | ND | ND | ND | ND | 0.05 | ND | ND | ND | ND | ND | ND | ND | ND |
| L7A_E11 | 0.16 | 0.05 | 0.16 | 0.11 | 0.22 | 0.22 | 0.22 | 0.27 | 0.22 | 0.27 | 0.32 | 0.22 | 0.22 | 0.22 | 0.32 | 0.11 | 0.48 | 0.27 | 0.38 | 0.48 | 0.86 | 0.16 | 0.48 | 0.59 | 0.32 | 0.59 | 0.16 | 0.59 | 0.43 | 0.54 |
| LE30 | 0.05 | 0.05 | 0.05 | 0.05 | ND | ND | ND | ND | ND | 0.05 | ND | ND | ND | 0.05 | ND | ND | ND | ND | 0.05 | ND | ND | 0.05 | ND | ND | ND | 0.05 | 0.05 | ND | ND | ND |
| *Massilia* | ND | ND | ND | ND | ND | ND | ND | ND | ND | ND | ND | ND | ND | ND | ND | 0.11 | ND | ND | ND | ND | ND | ND | ND | ND | ND | 0.05 | ND | ND | ND | ND |
| *Moryella* | 0.05 | 0.11 | 0.05 | 0.27 | ND | 0.05 | 0.38 | 0.22 | 0.27 | 0.11 | 0.05 | ND | ND | 0.05 | 0.22 | 0.22 | 0.05 | 0.11 | 0.27 | 0.11 | 0.05 | 0.27 | 0.59 | 0.16 | 0.43 | 0.05 | 0.11 | 0.16 | 0.22 | 0.05 |
| *Oscillospira* | 0.16 | ND | 0.32 | 0.38 | ND | 0.27 | ND | 0.05 | 0.11 | 0.43 | 0.11 | 0.05 | ND | 0.05 | 0.27 | 0.11 | 0.05 | 0.86 | 0.38 | 0.16 | 0.59 | 0.05 | 0.16 | 0.38 | 0.38 | 0.32 | 0.32 | 0.38 | 0.22 | 0.16 |
| p-75-a5 | 0.32 | 0.38 | 0.27 | 0.11 | 0.22 | 0.54 | 0.16 | 0.11 | 0.16 | 0.32 | 0.16 | 0.11 | 0.38 | 0.16 | 0.05 | 0.11 | 0.27 | 0.22 | 0.11 | 0.11 | 0.43 | 0.16 | 0.16 | 0.27 | 0.22 | 0.11 | 0.05 | 0.27 | 0.05 | 0.48 |
| *Paenibacillus* | ND | ND | ND | ND | ND | ND | ND | ND | ND | ND | ND | ND | ND | ND | ND | ND | ND | ND | ND | ND | ND | ND | 0.05 | ND | ND | ND | ND | ND | ND | ND |
| *Paludibacter* | 0.05 | 0.11 | 0.05 | 0.05 | 0.16 | 0.16 | ND | 0.16 | 0.11 | 0.05 | 0.05 | 0.05 | ND | 0.05 | 0.11 | 0.48 | 0.65 | 1.24 | 0.22 | 0.75 | 0.75 | 0.43 | 0.43 | 0.54 | 0.16 | 0.22 | 0.43 | 0.11 | 0.16 | 0.48 |
| *Pantoea* | ND | ND | 0.05 | ND | 0.05 | 0.05 | ND | ND | 0.05 | ND | 0.11 | ND | 0.11 | 0.05 | ND | 0.16 | 0.05 | ND | ND | 0.16 | ND | ND | 0.05 | ND | 0.05 | 0.16 | 0.11 | 0.05 | 0.11 | 0.05 |
| *Paracoccus* | ND | ND | ND | ND | ND | ND | ND | ND | ND | ND | ND | ND | ND | ND | ND | ND | ND | ND | ND | ND | ND | ND | 0.05 | ND | ND | ND | ND | ND | ND | ND |
| *Pedobacter* | ND | ND | ND | ND | ND | ND | ND | ND | ND | ND | ND | ND | ND | 0.05 | ND | 0.05 | ND | ND | ND | ND | ND | ND | ND | ND | ND | ND | ND | ND | ND | ND |
| *Peptostreptococcus* | ND | ND | ND | ND | ND | ND | ND | ND | ND | ND | ND | ND | ND | ND | ND | ND | ND | ND | ND | ND | ND | ND | ND | 0.11 | ND | 0.05 | ND | ND | ND | ND |
| *Polynucleobacter* | ND | ND | ND | ND | ND | ND | ND | ND | ND | ND | ND | ND | ND | ND | ND | ND | ND | ND | ND | ND | ND | ND | ND | 0.05 | ND | ND | ND | ND | ND | ND |
| *Prevotella* | 6.52 | 5.60 | 3.88 | 10.08 | 7.97 | 4.80 | 1.78 | 5.87 | 8.08 | 5.17 | 6.52 | 4.96 | 5.82 | 3.99 | 6.95 | 8.14 | 5.60 | 5.87 | 13.74 | 14.49 | 5.60 | 9.16 | 5.44 | 2.53 | 6.52 | 2.80 | 7.00 | 2.32 | 4.20 | 5.98 |
| *Pseudobutyrivibrio* | ND | ND | ND | ND | ND | ND | ND | ND | ND | ND | ND | ND | ND | ND | ND | ND | ND | ND | ND | ND | ND | ND | ND | ND | ND | ND | ND | ND | 0.05 | ND |
| *Pseudomonas* | ND | ND | ND | ND | ND | ND | ND | ND | ND | ND | 0.05 | ND | ND | ND | ND | 0.11 | ND | 0.11 | ND | 0.05 | ND | ND | ND | ND | ND | 0.05 | 0.05 | ND | ND | ND |
| Genus | Solid | | | | | | | | | | | | | | | Liquid | | | | | | | | | | | | | | |
|  | 3274 | | | 3292 | | | 3295 | | | 3298 | | | 3412 | | | 3274 | | | 3292 | | | 3295 | | | 3298 | | | 3412 | | |
|  | OP | OH | OP | OP | OH | OP | OP | OH | OP | OP | OP | OP | OP | OP | OP | OP | OH | OP | OP | OH | OP | OP | OH | OP | OP | OP | OP | OP | OP | OP |
| *Pyramidobacter* | ND | ND | ND | 0.11 | 0.05 | 0.05 | ND | ND | ND | 0.05 | ND | 0.05 | 0.05 | 0.11 | ND | 0.11 | 0.05 | 0.11 | 0.05 | 0.05 | ND | ND | ND | 0.27 | ND | 0.05 | 0.11 | ND | 0.05 | 0.16 |
| RFN20 | 0.05 | 0.59 | 0.11 | 0.11 | 0.43 | 0.05 | 0.05 | 0.54 | 0.05 | 0.16 | 0.27 | 0.05 | 0.43 | 0.22 | 0.22 | 0.43 | 0.65 | 0.05 | 0.48 | 0.65 | 0.27 | 0.38 | 0.54 | 0.38 | 0.22 | 0.43 | 0.54 | 0.22 | 1.02 | 0.27 |
| *Rhodococcus* | ND | ND | ND | ND | ND | ND | ND | ND | ND | ND | ND | ND | ND | 0.05 | ND | ND | ND | ND | ND | ND | ND | ND | ND | ND | ND | ND | ND | ND | ND | ND |
| *Ruminococcus* | 4.36 | 4.69 | 4.26 | 5.82 | 5.77 | 4.20 | 6.20 | 4.31 | 6.14 | 3.61 | 4.85 | 4.69 | 4.26 | 4.80 | 4.80 | 3.99 | 4.36 | 2.42 | 2.64 | 4.42 | 1.89 | 1.56 | 7.11 | 4.20 | 2.59 | 4.36 | 2.75 | 2.37 | 3.23 | 3.02 |
| *Selenomonas* | 0.86 | 0.27 | 0.32 | 2.96 | ND | 0.27 | 0.11 | 0.27 | 1.45 | 0.16 | 0.81 | 0.54 | 0.81 | 0.92 | 0.65 | 1.24 | 0.70 | 0.86 | 3.56 | 1.78 | 0.75 | 2.91 | 0.11 | 1.24 | 2.37 | 0.43 | 0.92 | 1.51 | 0.59 | 1.24 |
| *Sharpea* | ND | ND | ND | 0.16 | ND | ND | ND | ND | ND | ND | ND | ND | ND | ND | ND | ND | ND | ND | 0.05 | ND | ND | ND | ND | ND | ND | ND | ND | ND | ND | ND |
| SHD-231 | 0.05 | 0.05 | 0.05 | ND | ND | ND | 0.05 | ND | 0.05 | ND | 0.05 | 0.05 | 0.05 | ND | ND | ND | 0.22 | 0.11 | 0.05 | 0.05 | ND | 0.22 | 0.11 | 0.11 | 0.16 | 0.11 | ND | ND | 0.05 | 0.05 |
| *Solibacillus* | ND | ND | ND | ND | ND | ND | ND | ND | ND | ND | ND | ND | ND | ND | ND | ND | ND | ND | ND | ND | ND | ND | 0.05 | ND | ND | ND | ND | ND | ND | ND |
| *Sphingomonas* | ND | ND | ND | ND | ND | ND | ND | ND | ND | ND | ND | 0.05 | ND | ND | ND | ND | ND | ND | 0.05 | ND | ND | ND | ND | 0.22 | ND | ND | ND | ND | ND | ND |
| *Staphylococcus* | ND | ND | ND | ND | 0.05 | ND | ND | ND | ND | ND | ND | ND | ND | ND | ND | ND | 0.27 | ND | ND | 0.05 | ND | ND | 0.54 | ND | ND | ND | ND | ND | ND | ND |
| *Streptococcus* | ND | ND | ND | ND | ND | 0.05 | ND | 0.11 | ND | ND | ND | ND | ND | ND | ND | ND | 0.16 | ND | ND | 0.11 | ND | ND | 0.11 | ND | ND | 0.05 | ND | ND | ND | 0.05 |
| *Succinivibrio* | ND | ND | 0.05 | 0.27 | ND | 0.16 | ND | ND | 0.16 | ND | ND | ND | ND | 0.22 | ND | 0.27 | 0.16 | 0.16 | ND | 0.11 | 0.05 | 0.05 | 0.05 | 0.16 | 0.27 | 0.05 | 0.11 | ND | 0.16 | 0.22 |
| *Sutterella* | ND | ND | ND | ND | ND | ND | ND | ND | ND | ND | ND | ND | ND | ND | ND | ND | 0.11 | ND | ND | 0.27 | ND | ND | 0.05 | 0.05 | ND | ND | ND | ND | ND | ND |
| *Synergistes* | ND | 0.11 | ND | ND | ND | ND | ND | ND | ND | ND | ND | ND | ND | ND | ND | ND | ND | ND | ND | ND | ND | ND | ND | ND | ND | ND | ND | ND | ND | ND |
| TG5 | ND | ND | ND | ND | ND | ND | ND | ND | ND | ND | ND | ND | ND | ND | ND | 0.05 | 0.11 | ND | ND | 0.05 | ND | 0.05 | ND | ND | ND | ND | ND | 0.05 | 0.11 | 0.11 |
| *Treponema* | 0.05 | 0.54 | 0.05 | 0.11 | 0.48 | 0.05 | 0.16 | 0.32 | ND | 0.11 | 0.16 | 0.05 | 0.32 | 0.48 | 0.11 | 0.22 | 0.43 | 0.05 | 0.11 | 0.48 | 0.11 | 0.11 | ND | ND | 0.16 | 0.11 | 0.22 | 0.05 | 0.16 | 0.11 |
| unclassified | 66.54 | 75.43 | 74.46 | 66.11 | 74.25 | 75.75 | 76.83 | 76.35 | 68.05 | 73.49 | 70.20 | 68.16 | 64.71 | 64.39 | 68.16 | 67.13 | 77.21 | 77.05 | 58.73 | 66.33 | 76.99 | 73.60 | 75.70 | 75.43 | 70.53 | 70.96 | 71.98 | 71.39 | 69.29 | 71.66 |

**Table S5.** Least square means of community richness (CR) and community diversity (CD) values from 16S rRNA pyrotag sequencing of DNA isolated from ruminal contents of heifers fed an all-orchardgrass diet. ^a^

| Comparison | Group | CR_Chao | CR_Ace | | CR_Jackknife | | CD_Shannon | | CD_Simpson |  |
| --- | --- | --- | --- | --- | --- | --- | --- | --- | --- | --- |
| Diet Sequence ^b^ | OP/OH/OP | 488.98 | 555.47 | 619.67 | | 3.662 | | 0.1283 | | |
|  | OP/OP/OP | 509.95 | 605.34 | 790.45 | | 3.566 | | 0.088 | | |
|  | S.E.D.^c^ | 33.76 | 46.16 | 104.46 | | 0.097 | | 0.0474 | | |
|  | *P* > F | 0.466 | 0.290 | 0.114 | | 0.332 | | 0.404 | | |
|  |  |  |  |  | |  | |  | | |
| Phase | Liquid | 499.94 | 579.47 | 694.84 | | 3.784 ^d^ | | 0.1079 | | |
|  | Solid | 494.99 | 581.34 | 715.28 | | 3.444 ^e^ | | 0.1084 | | |
|  | S.E.D. | 30.19 | 41.28 | 93.43 | | 0.087 | | 0.0424 | | |
|  | *P* > F | 0.871 | 0.964 | 0.829 | | 0.0006 | | 0.991 | | |
|  |  |  |  |  | |  | |  | | |
| Source | Hay | 537.5 | 610.65 | 810.41 | | 3.801 ^d^ | | 0.0648 | | |
|  | Pasture | 457.64 | 550.15 | 599.71 | | 3.427 ^e^ | | 0.1515 | | |
|  | S.E.D. | 41.34 | 56.3 | 127.9 | | 0.119 | | 0.0581 | | |
|  | *P* > F | 0.065 | 0.294 | 0.112 | | 0.0041 | | 0.147 | | |

^a^ Bacterial communities as determined by 454 pyrosequencing of the V6-V8 16S rRNA gene. Estimators represent the average of 1000 iterations of subsampling to 1856 sequences per group.

^b^ OP/OH/OP: During three successive 28d-periods, heifers grazed pasture, then were fed hay, then grazed pasture. OP/OP/OP: Heifers grazed pasture during each of the successive 28d periods.

^c^ Standard error of the difference.

^d, e^ Means within comparison group differ (P<0.05).

**Legends to Supplemental Figures**

**Figure S1.** Scatter plot of the ordination points obtained from correspondence analysis of the automated ribosomal intergenic spacer analysis (ARISA) data matrix from the solid and liquid phases of ruminal digesta. Data points represent samples collected on the last 3 days of each of the 3 periods (P1-P3) from 5 heifers (n = 88; 44 from each phase, solid (S) and liquid (LIQ)). In this figure, the ordination points corresponding to the individual heifers were plotted separately to show the diet specific clustering of the ordination points [n = 18 for all heifers except heifer 3292 (n = 16)]. All the panels have the same scale. The 3 different symbols in each panel represent the ordination points corresponding to different periods. The open closed symbols represent the ordination points corresponding to SOL and LIQ phases, respectively, of ruminal digesta. Note the tight clustering of the ordination points for heifers 3298 and 3412 which remained on orchardgrass pasture throughout the periods compared to the diet-specific clustering observed for heifers 3274, 3292 and 3295 which were successively fed pasture, hay and pasture. ANOSIM R values for comparisons of pasture vs hay within each heifer and phase (with associated *P* in parentheses): 3274 LIQ, 0.306 (0.057); 3274 SOL, -0.003 (0.015); 3292 LIQ, 0.131 (0.007), 3292 SOL, 0.324 (0.003); 3295 LIQ, -0.021, (0.038), 3295 SOL, -0.262 (0.042).

**Figure S2.** Relative abundance of bacterial phyla observed in all heifers during all sampling periods by 454 pyrosequencing. OTUs were classified to the phylum level using the Greengenes database (DeSantis et al., 2006) with a consensus confidence threshold of 80 percent. OTUs from the liquid and solid phases from each heifer for each period were pooled to simplify the comparison. OTUs classified as phyla that were not present in all five heifers during every sampling period were condensed and are represented as “others”. Subsampling was performed to normalize among the samples. Regardless of heifer, or diet, the dominant classifiable phyla observed were Firmicutes, Tenericutes, and Bacteroidetes.
