# Supplementary figures and images for: Bacterial communities in the rumen of Holstein heifers differ when fed orchardgrass as pasture vs. hay
Source: Front Microbiol. 2014 Dec 9;5:689. doi: 10.3389/fmicb.2014.00689 (PMC4260508; doi:10.3389/fmicb.2014.00689)

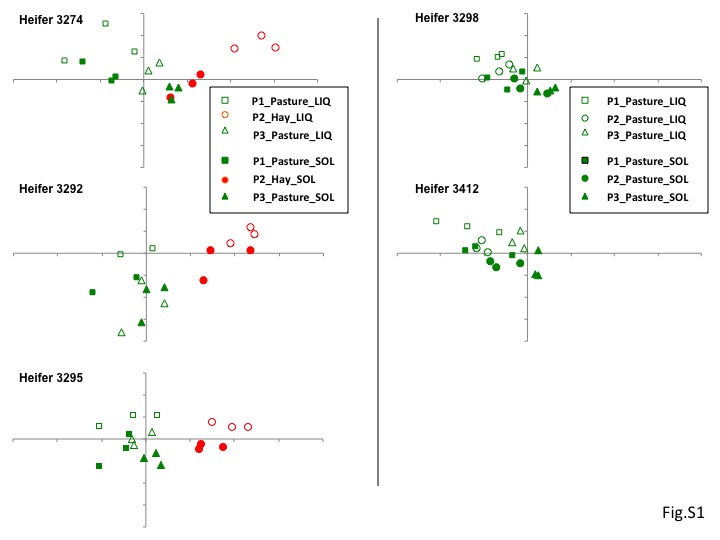

Supplement: Supplementary file 2 [file Image1.JPEG]

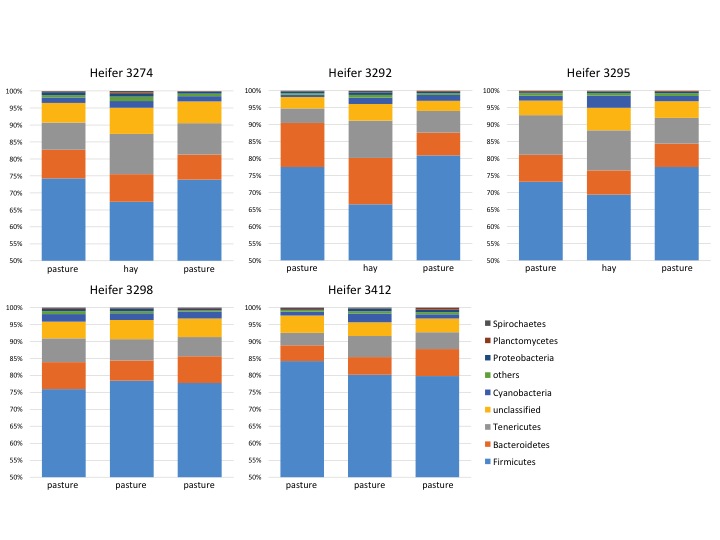

Supplement: Supplementary file 3 [file Image2.JPEG]
